# Methods Employed in Studies Identifying “Levels” of Test Anxiety in University Students: A Systematic Review

**DOI:** 10.3390/bs15030331

**Published:** 2025-03-07

**Authors:** Jerrell C. Cassady, Ser Hong Tan, Al Robiullah, Isabella Badzovski, Jessica Janiuk

**Affiliations:** 1 Department of Educational Psychology, Teachers College, Ball State University, Muncie, IN 47306, USA; al.robiullah@bsu.edu (A.R.); isabella.badzovski@bsu.edu (I.B.); jessica.janiuk@bsu.edu (J.J.); 2 Centre for Research in Child Development and Science of Learning in Education Centre, National Institute of Education, Nanyang Technological University, Singapore 637616, Singapore; serhong.tan@nie.edu.sg

**Keywords:** test anxiety, leveling strategies, systematic review, university students

## Abstract

Test anxiety research has been dominated by attention to theory building and examining the causes and consequences of this construct. However, recently, considerable attention has been turned toward using test anxiety as a diagnostic indicator of students who are at risk of underperforming in educational settings. This systematic review of the literature was focused on (a) describing the strategies used in the field, (b) highlighting the benefits and limitations of those approaches, and (c) offering guidance in creating a framework for appropriate methods when identifying severity levels on measures of test anxiety for university students. The results confirmed that the vast majority of studies on test anxiety have no formal “leveling” approaches (maintaining test anxiety as a continuous variable). However, when researchers do employ “leveling” strategies, the majority adopt inappropriate methods (e.g., single-sample splits). However, there are exemplars that demonstrate statistically sound procedures for identifying distinct profiles of test anxiety that may form a basis to build consensus around a classification method for elevated test anxiety.

## 1. Introduction

Test anxiety has been an area of vibrant research for over 50 years but has experienced a considerable increase in interest as assessments have become more prevalent in educational decisions regarding student progress, instructor efficacy, and institutional success (Lowe et al., 2011; von der Embse & Hasson, 2012). The research on test anxiety and learner experience has demonstrated compelling and recurring evidence that there are negative relationships among test anxiety and student performance (Putwain et al., 2016), the use of effective study strategies (Abdi et al., 2012; Thomas et al., 2018), and confidence or self-efficacy over academic evaluations (Cassady & Finch, 2020). Furthermore, test anxiety has been positively linked with negative affective variables including preclinical levels of depression, anxiety, and neuroticism (Cassady et al., 2019; Zeidner, 1998; Zeidner & Matthews, 2005), ratings of general well-being (von der Embse et al., 2013), and maladaptive coping strategies (Casari et al., 2014; Kamel, 2018; Putwain et al., 2016; Segool et al., 2013). While the general findings in the field support an overall negative relationship between test anxiety and academic success (Dawood et al., 2016), there is growing evidence that this representation may be oversimplified. That is, non-linear relationships tend to a provide superior model fit (Cassady et al., 2024), suggesting that low-to-moderate test anxiety may have a “facilitative” influence (Kader, 2016; Raffety et al., 1997).

The evidence of incidence rates for test anxiety has demonstrated that the reported rates of occurrence have been on the rise over the past 20 years. Reports in the early 2000s typically suggested that between 15 and 30% of students in university settings were likely to report experiencing test anxiety (Carter et al., 2008; McDonald, 2001; D. W. Putwain, 2007). However, recent analyses have identified that the reported rates of test anxiety have increased significantly in the past 10 years—with as many as 80% of university students reporting they experience moderate-to-severe levels of test anxiety (Cassady et al., 2023). The data also indicate that the reported level of test anxiety tends to increase across the educational timeline, with a higher proportion of university students reporting heightened test anxiety than students in elementary and secondary education settings (Putwain & Daly, 2014).

The dominant method for evaluating and assessing test anxiety has been self-report measures since Sarason and Sarason offered the Test Anxiety Questionnaire in 1952. Over that time frame, self-report instruments have almost universally relied upon using total scores or subscale values of componential aspects of test anxiety that meet the assumptions of continuity and normality as well as align with a theoretical model of test anxiety (Liebert & Morris, 1967; Putwain et al., 2020; Sarason, 1984; Spielberger, 1980). This approach to building the field of test anxiety research has bolstered several viable theoretical representations for the test anxiety construct, typically focusing on identifying the causes and consequences of test anxiety in traditional educational settings (von der Embse et al., 2018; Zeidner & Matthews, 2005). A less vibrant domain of research in the field (particularly for post-secondary students) has been focused on using these instruments to document the efficacy of interventions designed to relieve or mitigate the experience of test anxiety over time (Ergene, 2003; Hembree, 1988; Segool et al., 2013). In most of these intervention studies, there continues to be a primary focus on identifying success by measuring the “change” in measured levels of test anxiety on that continuous representation.

While theory development has been strong over the past half century, the recognition of increased rates of incidence and levels of severity in reported test anxiety among learners has created a groundswell of interest among education professionals, students, and parents to devote more attention to a practical orientation focusing on identification, intervention, and treatment efforts for learners who need support to reach optimal performance (see Hembree, 1988; Segool et al., 2013). This work is driven by awareness that interventions to support these learners in educational settings can increase student retention rates and promote greater degrees of student wellness and success (von der Embse & Hasson, 2012; Putwain et al., 2014). This work has been more prevalent in elementary through secondary school settings, perhaps driven by the greater focus of school support personnel to mitigate the social and emotional needs of learners. However, there is a growing interest in developing similar identification and intervention supports for learners in higher education settings, prompting our interest in reviewing the field to help form a consensus around effective methods to determine what students are “at risk” for academic difficulty due to test anxiety.

The purpose of this systematic review was to investigate studies in the literature that have employed categorical strategies for identifying “levels” of test anxiety in post-secondary populations. Using this corpus of the existing literature as a baseline, our goals are to identify existing trends and offer recommendations for best practices. Our choice of a systematic review, as opposed to other review methodologies (i.e., scoping review), was based on the primary purpose of the study to synthesize existing research in this population (Munn et al., 2018). Before identifying the trends in the field, we discuss the primary barriers to generating durable and valid categorical representations of test anxiety.

### 1.1. The Structure of Test Anxiety

Research in the field of test anxiety traditionally supported a two-factor representation, where “overall” test anxiety comprised two subordinate and related dimensional constructs. The classic terms for these subcomponents of test anxiety were Worry and Emotionality (Liebert & Morris, 1967), which have more recently also been discussed as “cognitive” and “physiological” dimensions (Cassady, 2023). Recent approaches to measuring test anxiety have also incorporated a “social” dimension of test anxiety (Friedman & Bendas-Jacob, 1997; Lowe et al., 2008) or made more fine-grained distinctions among aspects of test anxiety (e.g., Tension, Worry, Cognitive Interference, and Physiological Indicators; Putwain et al., 2020). Motivational constructs underlying the stressors faced in academic settings have also been proposed as critical determinants in the development or manifestation of test anxiety (e.g., Control–Value Theory; see Pekrun et al., 2011; Tan & Pang, 2023). These broader representations of multiple dimensions continue to support a more comprehensive view of contexts, histories, and beliefs of learners. However, there continues to be debate regarding which dimensions are distinct “types” of test anxiety and which are co-occurring psychological or ability constructs (see D. Putwain, 2025, for further discussion).

Research has routinely identified that the various dimensions of test anxiety are positively and significantly related to one another (Cassady, 2023; Hembree, 1988; Friedman & Bendas-Jacob, 1997; Lowe et al., 2008). However, the research also demonstrates differential influences of those factors on outcome variables such as student performance. Zohar’s (1998) “additive model” indicated that the cognitive and physiological dimensions exerted unique and negative impacts on performance. Subsequent research specified that the cognitive domain (e.g., Worry and Cognitive Interference) tends to be more directly tied to student performance declines, presumably due to the attention to barriers to effectively or efficiently encoding, storing, or retrieving the primary content either in the study or testing context (Cassady & Johnson, 2002; Cassady, 2023; Eysenck et al., 2007; D. Putwain, 2025). Another interpretation of the “combined” influence of cognitive and physiological indicators of test anxiety suggested that the physiological dimension serves as an “alert” mechanism, identifying a perceived threat in the academic setting, which subsequently activates the cognitive dimension (Hembree, 1988). In both of these representations, there is a tendency to focus on the cognitive aspects in a more “trait-like” representation (relatively stable over time and context) and the physiological aspect as more “state-like” (varied based on the task at hand; Cassady, 2023). Ultimately, most practitioners using assessment tools based on bifactor or multidimensional models of test anxiety tend to focus on “composite” or “total” scores to identify individuals with greater need; at the very least, this ensures that variations in manifestation are equitably included in the determination of test anxiety level (Friedman & Bendas-Jacob, 1997; Lowe et al., 2011; Sarason, 1984; von der Embse et al., 2013).

Naturally, variations in representation of the test anxiety construct as well as differences across measures in the discipline provide a clear challenge to establishing a universal agreement in determining clear “levels of test anxiety.” This is further exacerbated by the reality that there is no clinical definition for “test anxiety” accepted by professional psychologists and psychiatrists (e.g., American Psychiatric Association, 2022; World Health Organization, 1992). That is, the defining characteristics of test anxiety—as defined by theoretical models of test anxiety—are not clearly translated to defining a clinical disorder. A diagnosis of “clinical test anxiety” is typically made by clinicians through a combination of screening instruments and clinical interviews (Herzer et al., 2014). Furthermore, there is no established criterion for how prevalent test anxiety “should be” in the general population or what level of reported anxiety dictates elevated levels across measures (LeBeau et al., 2010; Warren et al., 1996). The absence of a “clinical sample” in studies has precluded the ability to perform a strong test of different models’ or measures’ precision in detecting truly detrimental levels of test anxiety (Herzer et al., 2014).

As such, researchers and practitioners who employ different measures of test anxiety are essentially examining different representations of the construct, creating a challenge for making reasonable comparisons across studies regarding the “levels” of test anxiety. A similarly troubling trend in the research is where measures that are not specifically designed to measure test anxiety are used to make conclusions about test anxiety. For instance, several studies over the years (Cardozo et al., 2020) have used the State-Trait Anxiety Inventory (STAI; Spielberger, 1989), to identify “levels of test anxiety” in their sample. Comparing those results to studies that employ another measure (e.g., the Test Anxiety Inventory, TAI; Spielberger, 1980) will likely generate incompatible representations of the populations, because the STAI is a “general anxiety” scale focused on trait and state subcomponents (not focused on testing), while the TAI focuses on the bifactor model of test anxiety (i.e., Worry and Emotionality over examinations specifically).

Our systematic review cannot hope to reconcile the variations of assessment instruments, and there is no intent to suggest that one assessment tool or theoretical model for test anxiety is preferred over others. The point of this review is to call attention to the factors that influence the variations in outcomes observed in the literature to guide researchers and practitioners alike to identify reasonable conclusions on identifying levels of severity for learners with test anxiety.

### 1.2. Measurement Challenges for Test Anxiety Scales

In addition to challenges imposed by varied assessment models for test anxiety and the imprecise definitions for “clinical levels” of test anxiety, traditional measurement issues also challenge this field of research. A fundamental assumption of most test anxiety scales is that the measure generates a total scale value across which incremental levels of test anxiety are measured in a relative interval fashion. This presumption does not necessarily align with classic assessment theory because most surveys use Likert-type items, which may not meet assumptions of interval measurement (DiStefano & Morgan, 2011). When directly tested, the scales generally demonstrate reasonable normality and consistency with expectations of interval measurement. The reality of the assessments is that they are based on a collection of responses to essentially ordinal data. Furthermore, studies examining differential item functioning (DIF) for test anxiety scales often report that not all items contribute equitably to the latent test anxiety construct (Baghei & Cassady, 2014; von der Embse et al., 2013), calling into question simple reliance on “total score” tabulation for determining a critical cut point. That is, validity threats are increased because a total scale score may misrepresent the latent test anxiety factor by undervaluing or overvaluing certain items.

A final historical barrier to the assessment of test anxiety has been the difficulties that often arise when instruments are composed of reverse-coded items or when inverting the valence of specific items in a scale. That is, some scales have items that focus on “high indication of anxiety” as well as those that are proposed to identify “low anxiety” with the direction to reverse-code and compile the values into a single factor (Sarason, 1984; Cassady, 2023). Work examining these approaches demonstrate that a reasonable factor solution may be derived, but the response patterns generally indicate that the reverse-coded items actually generate a secondary factor more representative of “test confidence” and should not be merely recoded and added to the total scale score (see Cassady, 2023; DiStefano & Motl, 2006).

### 1.3. Leveling Strategies

The overwhelming approach to research focuses on creating “level” attempts to identify “high” and “low” test anxiety groups within a population. The appeal of this approach beyond identifying learners with needs for support services is a simplified method of evaluating group differences (e.g., performance differences for high- and low-test-anxiety groups). Strategies employed for identifying groups of individuals based on the level or degree of a psychological construct typically fall into two broad methodological strategies. The first strategy (cf., “Expert-Based”) is dependent on experts indicating (a) the level of a construct needed to meet a “heightened” or “clinical” threshold and (b) how that threshold can be estimated by a given measure (Cizek & Bunch, 2007). As mentioned before, this is particularly difficult with test anxiety due to the absence of a clinical definition for the construct (Herzer et al., 2014). The second approach (“Data-Based”) employs data-driven methods that establish group membership based on response trends in the target population (Sarkin & Gülleroğlu, 2019).

#### 1.3.1. Expert-Based Methods

*Expert Ratings.* The expert-based methods operate under the assumption that an expert in the field can clearly identify that an individual exhibits symptoms consistent with a “clinical level” of that construct (Cizek & Bunch, 2007). One common strategy to accomplish this involves the Delphi Method, which involves experts indicating which items on a scale are most critical in identifying key diagnostic criteria as well as determining response levels that should be deemed clinically significant (Morgan et al., 2016). When the construct of interest has a clear clinical definition (e.g., Depression or Generalized Anxiety Disorder), this process is validated by comparing scores for a confirmed clinical population against a non-clinical population to demonstrate that the scale effectively differentiates (Cizek & Bunch, 2007). However, with no clinical definition for test anxiety, this final validation step is impossible (Sarkin & Gülleroğlu, 2019).

*Pre-determined cut scores.* Another strategy driven by expert judgments involves selecting a priori performance or rating levels on the measurement scale in question to determine levels of test anxiety. In a strategy similar to the Delphi Method, these expert-based judgments could be driven by clear theoretical alignment. However, the evidence is clear that pre-determined cut scores are often determined by arbitrary decisions based on scaling. An example of this approach to pre-determined cut scores is Campbell’s (2018) determination of levels of test anxiety using the Westside Test Anxiety Scale. Specifically, using the 5-point rating scale, students were deemed to represent five categories of test anxiety based on their average response to items falling into whole number ranges: 1.0–1.9 = Comfortably Low; 2.0–2.5 = Normal/Average; 2.5–2.9: High/Normal; 3.0–3.4 = Moderately High; 3.5–3.9 = High; and 4.0–5.0 = Extremely High test anxiety. These logical groupings are common across the field when a priori decisions are made, but without any empirical evidence that there is a clear differentiation among the identified levels, these findings are often unsupported or not validated.

#### 1.3.2. Data-Based Methods

Given the absence of an agreed-upon definition for “clinical” test anxiety, there is a significant preference for data-based strategies when establishing leveling criteria for test anxiety assessment tools. Data-based methods use patterns in respondent data to dictate group membership, varying widely in the levels of statistical sophistication and theoretical basis across studies and over the years. We recognize four general approaches that have dominated data-based approaches, including large-scale norming samples, “local sample” splits, item-focused group differentiation strategies, and person-centered group differentiation approaches.

*Large-scale norming samples.* The classic data-based approach to standard settings with assessment instruments involves gathering a large, representative sample with the target instrument and identifying the distribution of responses for the normal population. However, as many different areas of measurement theory have demonstrated, there are considerable barriers in this “basic” approach advocated in most classic test theory designs. First, developmental, cultural, racial, and gender variations in responses to individual items or in the distribution of total scores on assessment tools provide important reservations when performing these comparative analyses (Huntley et al., 2019). Given that there are commonly reported differences in test anxiety averages reported across age, gender, first-generation university student status, and race (Khan et al., 2015; D. W. Putwain, 2007; Putwain & Daly, 2014; von der Embse et al., 2018), separate norming tables would be required. Given these barriers, it is not surprising that few attempts have been made in this way. A notable exception to this trend for test anxiety measures exists with the Test Anxiety Inventory (Spielberger, 1980), but the norms established for that measure are over 40 years old, and comparative analyses have confirmed variations in the contemporary student performance on that scale (Szafranski et al., 2012).

*Local sample splitting.* A far more common approach to using a data-focused approach to form test anxiety groups involves using the sample in a single study as the source data. This approach has been particularly common in intervention studies that attempt to examine the impact of a treatment approach to relieve test anxiety and explore the differential utility of that intervention based on the level of test anxiety or use the test anxiety measure to identify those students who will receive the intervention.

These strategies may involve using percentile splits (e.g., median, tertile, or quartile) to differentiate among learners or the sample mean and standard deviation to generate a performance-based criterion (e.g., one standard deviation over the mean suggests “High Test Anxiety”) (Clinton & Meester, 2019; Myers et al., 2021). The obvious limitation to this broad approach is that the “cut scores” for each level of test anxiety will vary across studies, limiting comparability and generalization. This approach is also subject to flawed assumptions about the nature of test anxiety regarding the distribution of test anxiety in the population, the distinctiveness of the identified categories from one another, and the conceptualization of test anxiety itself (the measures chosen for a specific study will dictate the model of test anxiety assumed, which may vary greatly across studies).

*Scale- and item-focused group differentiation.* Two data-centric approaches that are primarily oriented toward response patterns on the given measurement tools for establishing profiles of test anxiety are Receiver Operating Characteristic curves (ROC) and the Rasch Rating Scale Method (RSM). These methods are both theoretically possible with test anxiety studies but are not commonly employed. The ROC method is consistent with traditional signal detection theory strategies that identify the rates of accurately identifying the presence of “the diagnosed condition” with an assessment instrument (taking into consideration rates of true and false positives and negatives) (Nahm, 2022). This method can be used to adjust the cut point on a measure that maximizes the number of “accurate” identifications. However, the notable limitation with using ROC for test anxiety is the requirement of a clinically identified subpopulation upon which those cut score decisions are validated. This is generally achieved in studies using ROC by using an expert judge to determine the “clinical group” (see von der Embse et al., 2021, for example).

The limiting factor imposed by the ROC’s need for a “clinical group” is overcome by the RSM, which also has the distinction of being a model focused on individual item characteristics rather than total scale performance levels. In a comparative study examining the accuracy of identifying individuals with ROC, RSM, and traditional standardized scores (e.g., z-scores), DiStefano and Morgan (2011) demonstrated that the results of RSM were as good or better than ROC and standardized test approaches, with the advantages of no required clinical sample and ability to estimate a “clinical” threshold for each individual student without being based on the sample population.

*Person-Centered Group Identification.* Ready access to statistical applications that support advanced modeling approaches has led to a sharp increase in the use of latent class analyses and cluster methodologies to identify learners with differentiable levels of expressed test anxiety (Pintado et al., 2016; Thomas et al., 2018). These strategies identify overall learner response patterns on one or more measures of test anxiety, identifying subgroups within the tested population who differ from one another in reliable ways. These analyses are largely isolated from preconceived biases in the expected number or composition of groups of test anxiety given the data-centric nature of the analyses.

## 2. Materials and Methods

The current study was designed as a systematic literature review exploring published peer-review research reporting analyses that either generated or relied upon subgroups of students based on their “level” of test anxiety. The focus of this investigation was limited to post-secondary students (i.e., university, college, and community college). The initial capture of articles for review occurred in April of 2022 and focused on a 10-year search (capturing all publications indexed with a publication year of 2012 or later). Delays in completing the work led to a re-examination of available articles in June 2024, augmenting the list to capture the period of time from 2012 to June 2024.

The search for relevant articles was conducted with support from a university librarian with expertise in framing large-scale record search and acquisition studies. Given the primacy of education and psychology in the research on test anxiety and the variations of language used to explain or discuss the construct, we employed multiple databases and search strings. The search databases used in this study were Academic Search Complete, APA PsycINFO, eBook Collection (EBSCOhost), ERIC, Web of Science, and the Psychology and Behavioral Sciences Collection (all searched in parallel). For search terms, to limit the overlap with other forms of anxiety, we were explicit in attempting to orient test, exam, or evaluation anxiety as the central construct but recognized the need to review both “anxiety” and “stress” as keywords that are common to the literature. The search terms used in all the databases included the following parameters, with a Boolean search string:

- Test AND (anxiety OR stress);
- Examination AND (anxiety OR stress);
- Evaluation AND (anxiety OR stress);
- For all searches, a limiter on the database for “university OR college” as the sample population was applied when enabled.

**Figure 1:**
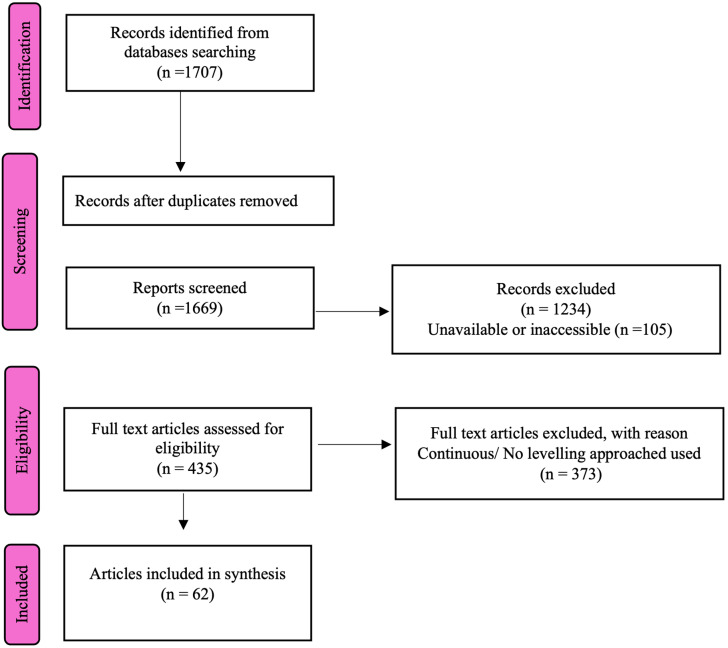
PRISMA table for review.

Once the searches were completed for all the databases, 1707 articles were identified for review (see Figure 1; Page et al., 2021). Initial screening of this list was conducted to eliminate duplicates captured in different databases due to variations in naming or citation. This cleaning of the datafile resulted in 1669 unique articles available for screening. The second stage of screening involved accessing full-text versions of the referenced studies and verifying that the study in question measured test anxiety (e.g., excluding review articles discussing test anxiety). This reduced the number of articles for review to 435 (1129 of the list did not directly measure test anxiety, and 105 were inaccessible in full-text format or only available in a language our team could not reliably translate).

The next step in the review process engaged in examining if the study in question used a “leveling” procedure. Of those 435 full-text studies reviewed, 373 did not employ a leveling procedure to the test anxiety measure. These studies primarily employed the measure of test anxiety as a continuous dependent variable (e.g., used in descriptive, correlational, or causal–comparative analyses) or a continuous predictor variable in a regression design. While these studies did not explicitly determine a standard to be reached as evidence of “high test anxiety”, the articles often used high values on a continuous measure being predictive of some outcome as impetus to suggest conclusions such as “students with high test anxiety were more likely to….”. While these studies were not the primary interest in this investigation, we classified them as “continuous” measure studies to capture this very common trend in the field and call attention to the trend in the field, mostly to alert readers to the tendency in the field to use the term “high test anxiety” without having a specific definition to support that terminology. To be sure, this should not be seen as a critique of that work. Indeed, research on the construct of test anxiety and how it interacts with other psychological or educational variables is often most effectively conducted when using a continuous measure of test anxiety. Among the myriad benefits of that approach is the ability to detect curvilinear relationships among test anxiety and related variables. However, given our focus on examining strategies employed to identify students with categorically differing levels of test anxiety, these studies lie outside our domain of inquiry.

Consequently, the review of articles resulted in 62 studies that measured test anxiety and documented different levels of test anxiety for the participants. These 62 studies are the primary focus in this investigation, but it is instructive to reflect upon the finding that with the large body of available published research on test anxiety in post-secondary learners over the past 12 years (n = 1669), only 26.1% (n = 435) were verifiable as empirical studies measuring test anxiety, and only 3.7% (n = 62) explicitly identified and explored test anxiety levels (see Figure 1). In short, there is a vibrant discussion about test anxiety in the literature, but there is relatively little research that directly examines the “level” of test anxiety.

Once there was agreement in the coding team that articles met the requirements of our targeted inquiry focused on using leveling procedures to identify groups of test-anxious students, each study was once again reviewed to identify the methodologies employed for leveling as well as the measures employed most commonly. Two independent coders examined each article and provided their interpretation of the reported method. The following list of five distinct categories was created among the team after reviewing the studies and was used in the independent ratings. It is important to note that we had a 6th category identified (item- or scale-based grouping) that was sought (i.e., RSM and ROC methods), but no studies using a university sample were identified to use that methodology.

- Continuous scale or no leveling: Articles that discuss test anxiety without specifying high or low levels of anxiety but may refer to trends related to high or low anxiety without specific criteria (e.g., regression analyses and path model studies). This was not a “leveling” strategy but was scored to identify the magnitude of this trend in the field.
- Local sample splits: Studies where group membership in the specific study was based on performance relative to that sample only (e.g., median, tertile, quartile splits, and mean ± standard deviation). These local sample splits typically created subgroups of similar sizes (as mandated by the reliance on only the specific study’s sample to determine groups).
- Published or validated splits: Articles that relied on pre-established and validated criteria to categorize participants’ test anxiety levels.
- Person-focused data-based grouping (e.g., “Cluster”): This approach relied on cluster methods, latent class analyses, profile analyses, and related strategies that employ student responses to classify people.
- Scale-based cut scores (“Logical Cut”): This approach establishes levels of test anxiety using a logical cut score based on scale values, typically without relying on the specific data from the study participants. Expert judgments often play a role in defining these scores

Cohen’s Kappa calculations were examined to ensure ratings were consistent (see Section 3 for Kappa values). Reconciliation for the few discrepancies in coding was achieved through discussion, with full agreement achieved.

## 3. Results

### 3.1. Leveling Strategies

Four distinct leveling strategies were identified in the studies captured in this review. Compared with related work in leveling research using psychological assessment instruments, these four strategies capture all the methods outlined except for (a) the expert-based method relying on comparison with clinical samples or the method of Delphi and (b) the data-based method using scale-focused group determinations (e.g., ROC and RSM). Naturally, the clinical comparison approach is limited by the absence of a clear clinical definition of test anxiety. As for the use of ROC and RSM, we are aware of studies using these strategies with younger populations (von der Embse et al., 2021) as well as with measures that are similar to—but not specifically—test anxiety (DiStefano & Morgan, 2011). However, when examining the classification of university students into groups based on their level of test anxiety, we found no examples of that approach being employed in the published literature, suggesting a viable domain of future research, perhaps by comparing modeling methods to see if there is convergence in group identification.

Below, the identified number of studies (and overall percentage) are provided with an explanation of the general strategies employed. For more details on specific studies and their identified classification level, please refer to Table A1. It is instructive to clarify that the studies below have been classified by the instrument that was used in the study to “create” the different levels of anxiety. In some cases, multiple measures of test anxiety were collected, but the attention in the sorting task in this study was focused on the method used to establish a leveling decision. Those studies that employed multiple scales generally used the secondary scale as a validation check (i.e., levels established with Measure A generated group means that were significantly different on Measure B).

*Scale-based cut scores (“Logical Cut”).* The most commonly identified strategy used to identify levels of test anxiety in this review used a “scale-based cut score” approach based on an arbitrary criterion process that did not employ data-driven procedures to establish levels of test anxiety (n = 31, 50% of leveling studies). These cut scores were generally established based on the scale values for a measure, using a logical criterion that was not informed or influenced by the responses of the participants in the study in question. These may be characterized in many cases as involving or including expert judgments as well, as the researchers engaged in the process of determining the cut scores were clearly experts in either data analyses or test anxiety as a construct. As mentioned before, Campbell (2018) employed this method with the Westside Test Anxiety Scale. Another example of this approach was carried out by the authors Zargarzadeh and Shirazi (2014), who reported using student responses to 25 true–false items based on Sarason’s anxiety measure (score range = 0–25). They offered that values of less than 12 indicated minor anxiety, 12–20 demonstrated moderate anxiety, and greater than 20 counted as severe.

Evaluation of the inter-rater reliability for identifying studies as fitting into this category employed Cohen’s Kappa, which resulted in a value of 0.76 (indicating a strong level of agreement). A review of misaligned classifications indicated that those errors were generally due to classifying these studies as using sample-based splitting strategies (which will be described next).

*Local sample splits (“Splits”).* Another common approach was to employ sample splitting techniques using only the participants for the study in question (n = 10, 16.1% of leveled studies). These strategies include creating median, tertile, or quartile splits (generating equivalent subgroups) or using the mean and standard deviation of the study sample to identify extreme groups. In contrast to the scale-based cut scores, the sample splitting strategies are generally used with no attention to the potential range of responses. Cohen’s Kappa for this group of studies was 0.68, with initial disagreement primarily occurring with the scale-based cut scores.

*Person-focused data-based grouping (“Cluster”).* A qualitatively different method of using a study sample to determine different levels of test anxiety relies on clustering, latent class, latent profile, or other statistically derived group classification strategies (n = 10, 16.1% of leveled studies). These approaches differ from the sample splitting methods in that the groups identified through clustering, latent class, or profile analyses focus primarily on identifying data-derived differences among the respondents in a sample. As such, group membership is independent of the potential range of the scale, the number of groups is determined empirically, and the distribution of the sample among the groups is not pre-determined with respect to size or percentage of the population. Our team of independent coders had a Cohen’s Kappa agreement value of 1.0, indicating full agreement in initially classifying the studies using latent classification strategies.

*Standardized or published criteria (“Validated”).* The final defined method of establishing groups based on levels of test anxiety identified in the sample was the use of pre-determined severity standards that were previously established through empirical strategies (n = 11, 17.8% of leveled studies). That is, these studies made use of a previously validated data-driven strategy for identifying learners at various levels of test anxiety. This approach could be characterized as having the highest level of external validity (among those identified in our review) because the grouping is based on a sample other than the one in the current study. This strategy also does not have a predetermined or sample-based limitation in the number of respondents who will be classified at various levels of test anxiety.

However, it is critical to recognize that the previously determined severity standards referenced in these 11 studies are also subject to limitations. That is, if the source criteria used were based on limited samples or inappropriate methodological approaches, a ripple effect in misclassification could occur. Cohen’s Kappa for rater agreement in identifying studies using this approach was 0.88, demonstrating high agreement.

### 3.2. Use of Test Anxiety Measures

To help summarize the overall representation of the field, our team also examined the specific measures used in the field to explore leveling strategies. As demonstrated in Table 1, the Test Anxiety Inventory was the most commonly used scale in the last 12 years when researchers were seeking to identify different “levels” of test anxiety. While the TAI has validated existing identified levels (Spielberger, 1980), not all studies in our review used those criteria (as shown, both logical cut and validated strategies were used). There is no clear evidence that the standards established in 1980 are still relevant to the current population (see Szafranski et al., 2012). Collectively, a review of Table 1 illustrates the diversity of measures as well as methods for determining the severity levels of test anxiety reported by participants in post-secondary settings. A more specific review of the 62 studies that provided specific leveling data is provided in Table A1, which can be used to identify the specific studies that employed the various methods of leveling for the interested researcher or practitioner.

## 4. Discussion

This systematic review of studies employing “leveling strategies” in university samples was undertaken to provide a documentation of the strategies that have been used in the field to date as well as provide recommendations to support more valid and consistent methods for identifying and subsequently supporting individuals in post-secondary education settings. Prior research focused on the influence of test anxiety has repeatedly demonstrated that university students report high levels of test anxiety (Cassady, 2023) and that there are durable and reliable negative relationships between test anxiety and students’ study habits, coping strategies, and academic performance when levels of anxiety are elevated. This body of research has demonstrated reliable predictive power for identifying learners who are more likely to underperform as well as withdraw from university settings. Our focus in this work is to provide a pathway to identification and intervention such that early indicators of high test anxiety or poor academic readiness indicators can be detected early in the university experience to connect learners with interventions that will promote student retention and success (Cassady et al., 2022).

### 4.1. Limited Attention Identifying “Elevated” Levels

The results of our review of the literature initially demonstrated a considerably low amount of explicit attention to true “leveling” in the field. That is, only 62 of the 435 studies that explicitly measured test anxiety in learners in post-secondary settings (14.3%) in the past 12 years were identified in our review of the literature to use direct leveling strategies to identify different proposed “groups” of students based on their level of test anxiety. The remaining 373 studies largely employ test anxiety as a continuous variable, typically examining the level of test anxiety in a regression model or as the outcome variable of interest. As the first point of interest, we raise this as a possible domain of deeper inquiry in the field. There is considerably more attention to identification and intervention efforts in students from Grades 1–12 (see Tan et al., in press), despite evidence that the severity and prevalence of test anxiety tends to increase as students progress into university (Cassady, 2023; Putwain et al., 2010; von der Embse & Witmer, 2014). We anticipate that this gap in the focus on identifying and serving students will rapidly decrease as universities continue to find methods to better serve and subsequently retain more students who arrive with less developed study habits and strategies that are known to boost success in post-secondary settings. While there is great theoretical benefit to learning how test anxiety influences learners’ outcomes in educational settings by exploring test anxiety as a continuous measure, for the simplicity of diagnosis often sought in programs designed to identify and serve “at risk” students, continued efforts to building a base of empirically driven strategies for classifying learners with differing levels of test anxiety shows great promise. However, as outlined next, we assert that careful attention to the methods of forming these classification groups is critical to building a consensus on what constitutes a clinical need in test anxiety.

### 4.2. Methodological Recommendations for Identifying “Levels” of Test Anxiety

From a methodological perspective, our primary finding is that the bulk of work that makes use of a leveling strategy for test anxiety has been employing questionable strategies. That is, the overwhelming majority of studies employed two strategies that we propose are not preferred for building a disciplinary approach to identify a meaningful criterion for defining “elevated” or “high” test anxiety among post-secondary students. The two most common strategies employed arbitrary cut scores and “local splits” to determine levels. The measurement concerns with both of these strategies are clear and have been established in the field for years (Cizek & Bunch, 2007; Sarkin & Gülleroğlu, 2019). Put simply, the result for studies that use local splits is merely an identification of the “most test anxious people in the current sample”, which will vary from study to study. For the studies that employ an arbitrary cut score (usually using the average value for each item, e.g., Campbell, 2018), the risks are based on the potential that each item does not have equitable value in determining the underlying construct or that the cut points are not realistically aligned with any indication of severity. For the latter strategy, the absence of an explicit definition or clinical criterion to validate the estimated cut scores further hampers the ability to establish meaningful validity.

This overarching limitation in the vast majority of studies poses a risk to having an established criterion for determining levels of severity for test anxiety and subsequently calls into question any reported estimates of the incidence of test anxiety in the university population. That is, while researchers have identified projections of the percentage of students who report moderate to high levels of test anxiety (Cassady et al., 2023), these estimations are only relevant within the context of the measure in use and have to be reviewed within the context of the measures employed as well as the methods of labeling levels of test anxiety.

However, rather than simply projecting a negative narrative about using test anxiety measures to identify levels of need in the post-secondary student population, we see a key opportunity to build consensus in defining “high test anxiety” as a criterion-based characteristic that clearly identifies individuals who have a measured level of test anxiety that puts them at risk for learning loss in the university setting. The evidence from the few studies that have employed person-centered strategies (e.g., cluster methodology and latent-class analyses) demonstrate that there are measurable differences among learners in higher education that can be used to identify clear cut scores that indicate qualitatively different perceptions of threat or stress in academic assessments (Lin, 2021; Thomas et al., 2018). To that end, we propose that research with a focus on identifying learners with levels of test anxiety that are likely to lead to academic challenges (either through performance or inappropriate study strategies and coping behaviors) should employ a few basic minimum strategies.

*Recommendation 1: Reduce the Use of Local Splits.* Our first recommendation is to eliminate the simplified method of using a local sample to set the “standards” for determining those with and without elevated test anxiety. Aside from the inherent changes that arise whenever you use a local and changing sample to identify “high test anxiety”, using local splits reduces the ability to compare the efficacy of interventions across settings. Intervention methods that prove highly useful in one setting may be less effective when the strategy for identifying the recipients of that intervention approach differs. Furthermore, the data have repeatedly demonstrated that there is usually a disproportionate number of learners who fall into the different categories of test anxiety (see Cassady, 2023; Putwain et al., 2016), but local sample splits almost invariably assign even numbers of students to their different “levels”. The only exceptions to this recommendation arise when programs are in the initial phases of attempting to identify their high-need population with a new assessment process or when resources are dramatically limited (and only a very small proportion of students can be supported).

*Recommendation 2: Eliminate Arbitrary Cuts.* Our recommendation about arbitrary cut scores is more extreme based on our experience reviewing the field. A prototypical arbitrary cut process involves identifying the range of potential responses and then dividing the level of severity based on the original response scale options. For instance, if a 4-point scale is used, the “high anxiety” group would often be identified by anyone who averages 3.5 on that 4-point scale overall. Setting logically based cut scores simultaneously makes weak items overly important and strong items undervalued in the identification of the psychological construct (Cizek & Bunch, 2007; Sarkin & Gülleroğlu, 2019). These logical splits were traditionally valuable, but in the current era of ready access to more effective statistical solutions to test the relative utility of each item in determining the total scale, these approaches are simply outdated.

*Recommendation 3: Adopt and Adapt.* Overall, our suggestion for carrying out this leveling work in the field is to build upon existing and validated approaches with existing scales (with the explanation that this does not mean replicating cut scores identified in the prior two recommendations). When determining the viability of adopting a prior finding, we recommend that the method used in the original study should have been based on a representative or generalizable population (relevant to the population in question) and that the conditions of establishing the criterion scores can be replicated and validated. With the studies reported in this systematic review, the most promise was shown for the cluster and latent class (person-centered strategies). With sufficient sampling, these strategies allow a greater ability to clearly differentiate among learners to provide categorical identifiers for levels of test anxiety. An extension of this strategy that has not been identified in the current literature is to replicate these latent grouping strategies for subgroups of students (e.g., first-generation learners or racial or ethnic minority groups) to build a more comprehensive predictive identification of the level of test anxiety that should be targeted as the “cut score” for providing outreach and intervention services to learners with different backgrounds or experiences.

Finally, while the availability of clustering and latent class procedures shows great promise for identifying levels of test anxiety, these are not necessarily the optimal approaches. As mentioned, there were no identified studies in our review that used individual item characteristics to determine cut scores for levels of anxiety for post-secondary learners. However, prior research with younger populations shows that these strategies (e.g., ROC and RMS; see von der Embse et al., 2021) have a great advantage in ensuring that the most relevant items or subscales in a measurement tool contribute the most to the final determination of the criterion for identifying learners’ levels of test anxiety.

A final note on this recommendation is to highlight that we recommend “Adopt and Adapt”; that is, merely adopting existing values from prior studies is not necessarily the optimal solution for building a diagnostic procedure in an academic context where the goal of the institution is to identify learners with the greatest needs for intervention related to test anxiety supports. As the student body differs from the original study location and the new implementation location, adjustments may be required. As such, employing a validation check periodically is critical. To reiterate, while the TAI was the most commonly used measure for leveling studies in our review, those that employed the published criteria for “high test anxiety” were based on the 1980 norms, which are certainly out of touch with the contemporary post-secondary student population.

### 4.3. Limitations and Future Directions

This systematic review is limited in the ability to reliably offer a “solution” for defining “clinical” test anxiety or setting the standard for determining what level or degree of test anxiety should be considered detrimental for learners. However, the primary purpose of this study was to establish the “field as it is”, which hopefully will enable the community of scholars focused on this work to take on the next step—identifying a common definition for elevated or clinical levels of test anxiety. The primary limitations we observed in this process are the diversity of assessment measures, the variations in leveling strategies used in prior work, and the wide variations in student populations addressed in this work. Our observation across the post-secondary population in this work has revealed an unexpected lag in the research with this population. That is, there is a far lower degree of attention to establishing “levels” of test anxiety for university students to identify high test anxiety for support and treatment than for younger populations (i.e., children and adolescents; see Tan et al., in press). This is an area of growth for the field that appears in line with the current needs in higher education retention goals. As more students are attending post-secondary training, the number of individuals who are likely to feel stressed or overwhelmed by test anxiety is increasing. If institutions of higher education intend to support these students (if for no other reason than to maintain enrollment levels), employing diagnostic assessments to identify learners with heightened needs is one avenue for institutional priorities as well as a vibrant area for program efficacy research (see Cassady, 2022, for an extended discussion).

## Figures and Tables

**Table 1:** Test anxiety measures and methods of leveling.

Test Anxiety Instrument	Number of Studies	Split	Val	LogCut	Cluster
China Language Anxiety Survey (CLAS)	1	-	-	🗸	-
Cognitive Test Anxiety Scale (CTAS)	5	🗸	🗸	-	🗸
Foreign Language Classroom Anxiety Scale (FLCAS)	6	🗸	-	🗸	-
German Test Anxiety Inventory (PAF and PAF-E)	4	-	🗸	-	-
Hamilton Anxiety Scale	1	-	🗸	-	-
Medical Student Stressor Questionnaire (MSSQ)	1	-	-	🗸	-
Motivated Strategies for Learning Questionnaire (MSLQ)	1	-	-	-	🗸
One-Off Scales Created by the Researcher for the Current Study	4	🗸	-	🗸	🗸
Sinha Anxiety scale	1	-	-	🗸	-
State-Trait Anxiety Inventory (STAI)	4	-	🗸	🗸	-
Test Anxiety Inventory (TAI)	14	-	🗸	🗸	🗸
Test Anxiety Questionnaire (TAQ)	3	🗸	-	🗸	-
Test Anxiety Scale (TAS)	6	-	-	🗸	🗸
The Express Test–The Diagnostics of Examination Anxiety	1	-	-	🗸	-
Visual Analog Scale (VAS)	1	🗸	-	-	-
Westside Test Anxiety Scale (WTAS)	8	🗸	🗸	🗸	-
Zung Self-Rating Anxiety Scale	1	-	-	🗸	-

Notes: Identified leveling methods: Cont—test anxiety measured as continuous variable, no leveling. LogCut—logical cut scores, not data-driven. Val—validated, using standardized methods or published leveling values. Splits—data-based splits using “local” samples’ median, mean, etc. Cluster—student-focused grouping methods.

## Data Availability

Data are contained within the article and Appendix A.

## Appendix A. Supplemental Materials for Reviewed Studies

**Table A1:** Leveling Study Extended Details.

Citation	Sample Location	Sample Size	Test Anxiety Scale(s)	Leveling Strategy
Ramezani et al. (2016)	Iran	98	Sarason Test Anxiety Scale	LogCut
Fathi et al. (2017)	Iran	250	Test Anxiety Inventory (TAI)	LogCut
Ahmad et al. (2018)	Pakistan	126	Westwide Test Anxiety Scale	LogCut
Akkakoson (2016)	Thailand	88	Modified Foreign Language Classroom Anxiety Scale (MFLCAS)	LogCut
Ali et al. (2015)	Pakistan	387	The Westside Test Anxiety Scale (WTAS)	LogCut
Al-Khasawneh (2016)	Saudi Arabia	97	Foreign Language Classroom Anxiety Scale (FLCAS)	LogCut
Andujar and Cruz-Martínez (2020)	Spain	176	Cognitive Test Anxiety Scale Revised (CTAS-2)	Cluster
Apostolidis and Tsiatsos (2021)	Greece	40	State-Trait Anxiety Inventory (STAI) Pasco Hernando Community College (PHCC) test anxiety questionnaire Neurosky Device	LogCut
Arain (2021)	Saudi Arabia	150	Test Anxiety Questionnaire (TAQ)	LogCut
Arici (2018)	Turkey	46	The anxiety inventory developed by Necla Öner and Deniz Albayrak	LogCut
Atasheneh and Izadi (2012)	Iran	60	Foreign Language Class Anxiety Scale (FLCAS)	LogCut
Basith et al. (2019)	China	250	China Language Anxiety Survey (CLAS)	LogCut
Bojović (2020)	Serbia	60	Foreign Language Classroom Anxiety Scale	Split
Campbell (2018)	US	48	Westside Test Anxiety Scale (WTAS)	LogCut
Cardozo et al. (2020)	Brazil	56	State-Trait Anxiety Inventory (STAI)	LogCut
Casari et al. (2014)	Argentina	140	Cognitive Test Anxiety Scale	Split
Chan and Bauer (2016)	US	164	Motivated Strategies for Learning Questionnaire (MSLQ)	Cluster
Clinton and Meester (2019)	US	111	Cognitive Test Anxiety Scale (CTAS)	Split
Dawood et al. (2016)	Kingdom of Saudi Arabia	277	Test Anxiety Inventory (TAI)	LogCut
Dimitriev et al. (2016)	Russia	96	State Trait Anxiety Inventory-State Anxiety Subscale	LogCut
Eddington et al. (2012)	US	30	Zung Self-Rating Anxiety Scale	LogCut
Farnia et al. (2017)	Iran	196	Test Anxiety Inventory (TAI) --> it’s the TAS (Sarason) though the authors called it the TAI	Validated
Fernández-Castillo and Caurcel (2019)	Spain	511	Spanish version of the State-Trait Anxiety Inventory (STAI)-state-anxiety scale	Validated
Gargabi et al. (2014)	Iran	638	Test Anxiety Inventory	LogCut
Hedlefs-Aguilar et al. (2021)	Mexico	140	36 experimental vignettes	Cluster
Hewitt and Stephenson (2012)	Spain	40	Foreign Language Classroom Anxiety Scale (FLCAS)—with a text anxiety subscale	Split
Higham et al. (2021)	UK	264	test anxiety questionnaire	LogCut
John Lothes et al. (2021)	US	43	Test Anxiety Inventory	Validated
Kausar et al. (2018)	Pakistan	60	MSSQ	LogCut
Khan et al. (2015)	India	50	Sinha Anxiety scale (Sinha W.A. Self Analysis form)	LogCut
Khoshhal et al. (2017)	Saudi Arabia	111	Visual analog scale (VAS) and twelve questions	Split
Klug et al. (2021)	Germany	202	The test anxiety questionnaire (PAF)	Validated
Knoll et al. (2019)	US	301	Revised Test Anxiety Scale & Test Anxiety Inventory-5	Validated
Kusturica et al. (2019)	Bosnia	210	Westside Test Anxiety Scale (WTAS)	LogCut
Manchado Porras and Hervías Ortega (2021)	Spain	201	An adaptation of the German Test Anxiety Inventory (TAI-G)	Split
Minihan et al. (2021)	Iran	60	Test Anxiety Inventory (TAI)	LogCut
Morales-Martínez et al. (2021)	Mexico	474	12 experimental scenarios	Cluster
Myers et al. (2021)	US	173	Trait Test Anxiety Inventory (T-TAI)	Split
Ne’Eman-Haviv and Bonny-Noach (2019)	Israel	814	Test Anxiety Questionnaire (TAQ)	Split
O’Donnell (2017)	US	284	Test Anxiety Inventory (TAI)	Validated
Oflaz (2019)	Turkey	61	Foreign Language Classroom Anxiety Scale (FLCAS)	LogCut
Palkar et al. (2021)	India	114	Test Anxiety Scale (TAS)	LogCut
Patience and Chinyere (2014)	Nigeria	41	Test Anxiety Meter (modified)	LogCut
Pintado et al. (2016)	Spain	94	Test Anxiety Inventory (TAI), Test Anxiety Scale (TAS), The Inventory of Test Anxiety (ITA)	Cluster
Qin et al. (2021)	China	300	14-item Hamilton Anxiety Scale (HAMA-14)	Validated
Rajiah and Saravanan (2014)	Malaysia	225	Westside Test Anxiety Scale (WTAS)	Validated
Relojo-Howell and Stoyanova (2019)	Bulgaria	151	Westside Test Anxiety Scale (WTAS) & Cognitive Test Anxiety (CTA)	LogCut
Sa’ad Alzboon (2016)	Jordan	354	Developed Own Scale of 48 Items	LogCut
Sarkin and Gülleroğlu (2019)	Turkey	290	Test Anxiety Scale (Spielberg)	Cluster (multiple strategies)
Savytska (2019)	Ukraine	72	The Express Test—The Diagnostics of Examination Anxiety	LogCut
Schweden et al. (2020)	Germany	38	German Test Anxiety Inventory (GTAI-A)	Validated
Shakil and Saad Ahmed (2019)	Pakistan	200	Westside Test Anxiety Scale	LogCut
Thomas et al. (2018)	US	807	Cognitive Test Anxiety Scale–Revised MSLQ–Test Anxiety (MSLQ-TA) subscale FRIEDBEN Test Anxiety Scale (FTAS)	Cluster
Vaz et al. (2018)	India	341	Test Anxiety Scale (developed by Manipal Academy of Higher Education )	LogCut
Warnecke and Krohne (2019)	Germany	40	Test Anxiety Inventory (TAI)	Validated
Woldeab and Brothen (2019)	US	631	Westside Test Anxiety Scale (WTAS)	Split
Zhang and Qin (2020)	China	210	Test Anxiety Inventory (TAI)	LogCut
Lin (2021)	Taiwan	368	questionnaire designed by the researchers to measure statistics anxiety	Cluster
Zargarzadeh and Shirazi (2014)	Iran	94	Sarason Test Anxiety Scale	LogCut
Zavery et al. (2021)	Switzerland	16	Prüfungsangstfragebogen (PAF, test anxiety questionnaire)	Validated
Guo et al. (2024)	China	64	Test Anxiety Inventory (TAI)	Splits
Thomas and Ozer (2024)	US and Turkey	422	CTAS	Validated

Note. Identified leveling methods: LogCut—Logical Cut Scores, not data-driven. Val—Validated, using standardized methods or published leveling values. Splits—Data-based splits using “local” samples’ median, mean, etc. Cluster—Student-focused grouping methods.

Supplemental References List (Reviewed Leveling Studies)

(Ahmad et al., 2018) Ahmad, N., Hussain, S., & Khan, F. N. (2018). Test anxiety: Gender and academic achievements of university students. *JPMI: Journal of Postgraduate Medical Institute*, *32*(3).
(Akkakoson, 2016) Akkakoson, S. (2016). Speaking anxiety in English conversation classrooms among Thai students. *Malaysian Journal of Learning and Instruction*, *13*(1), 63–82.
(Ali et al., 2015) Ali, M., Asim, H., Edhi, A. I., Hashmi, M. D., Khan, M. S., Naz, F., Qaiser, K. N., Qureshi, S. M., Zahid, M. F., & Jehan, I. (2015). Does academic assessment system type affect levels of academic stress in medical students? A cross-sectional study from Pakistan. *Medical Education Online*, *20*, 27706. https://doi.org/10.3402/meo.v20.27706.
(Al-Khasawneh, 2016) Al-Khasawneh, F. M. (2016). Investigating foreign language learning anxiety: A case of Saudi undergraduate EFL learners. *Journal of Language and Linguistic Studies*, *12*(1), 137–148.
(Andujar & Cruz-Martínez, 2020) Andujar, A., & Cruz-Martínez, M. S. (2020). Cognitive test anxiety in high-stakes oral examinations: Face-to-face or computer-based? *Language Learning in Higher Education*, *10*(2), 445–467.
(Apostolidis & Tsiatsos, 2021) Apostolidis, H., & Tsiatsos, T. (2021). Exploring anxiety awareness during academic science examinations. *PLoS ONE*, *16*(12), e0261167. https://doi.org/10.1371/journal.pone.0261167.
(Arain, 2021) Arain, F. R. (2021). Does test anxiety and gender have an impact on OSCE performance among medical students? *Middle East Journal of Family Medicine*, *19*(11), 101–107. https://doi.org/10.5742/MEWFM.2021.94165.
(Arici, 2018) Arici, I. (2018). The relationship between the music teacher candidates’ computer-assisted teaching attitudes and exam anxiety in computer literacy. *Journal of Education and Training Studies*, *6*(11), 215–222.
(Atasheneh & Izadi, 2012) Atasheneh, N., & Izadi, A. (2012). The role of teachers in reducing/increasing listening comprehension test anxiety: A case of Iranian EFL learners. *English Language Teaching*, *5*(3), 178–187.
(Basith et al., 2019) Basith, A., Musyafak, N., Ichwanto, M. A., & Syahputra, A. (2019). Chinese learning anxiety on foreign students. *European Journal of Educational Research*, *8*(4), 1193–1200.
(Bojović, 2020) Bojović, M. (2020). ESP speaking strategies and foreign language anxiety in higher education classroom. *Strategije Usmenog Izražavanja I Anksioznost U Učenju Engleskog Jezika Struke U Visokoškolskoj Nastavi*, *45*(5), 181–203. https://doi.org/10.19090/gff.2020.5.181-203.
(Chan & Bauer, 2016) Chan, J. Y., & Bauer, C. F. (2016). Learning and studying strategies used by general chemistry students with different affective characteristics. *Chemistry Education Research and Practice*, *17*(4), 675–684.
(Dimitriev et al., 2016) Dimitriev, D. A., Saperova, E. V., & Dimitriev, A. D. (2016). State anxiety and nonlinear dynamics of heart rate variability in students. *PLoS ONE*, *11*(1), e0146131. https://doi.org/10.1371/journal.pone.0146131.
(Eddington et al., 2012) Eddington, A., Mullins, L., Byrd-Craven, J., & Chaney, J. (2012). An experimental examination of stress reactivity in adolescents and young adults with asthma. *Children’s Health Care*, *41*(1), 16–31. https://doi.org/10.1080/02739615.2012.643287.
(Farnia et al., 2017) Farnia, V., Salimi, Y., Alikhani, M., & Mohammadi, A. (2017). The mediating role of emotional intelligence in coping strategies and test anxiety in students of Kermanshah University of Medical Sciences, Kermanshah, Iran in 2013–2014. *Iranian Journal of Psychiatry & Behavioral Sciences*, *11*(4), 1–6. https://doi-org.proxy.bsu.edu/10.5812/ijpbs.9254.
(Fathi et al., 2017) Fathi, A., Barmehziar, S., & Mohebi, S. (2017). Survey test anxiety in pre-university students in Qom and its related factors in 2016. *Education Strategies in Medical Sciences*, *10*(4), 270–276.
(Fernández-Castillo & Caurcel, 2019) Fernández-Castillo, A., & Caurcel, M. J. (2019). Autoestima, horas de sueño y ansiedad-estado ante exámenes académicos [Self-esteem, hours of sleep and state-anxiety before academic tests]. *Revista Argentina de Clínica Psicológica*, *28*(4), 348–355.
(Gargabi et al., 2014) Gargabi, Z. H., Nouri, R., Moradi, A., & Sarami, G. (2014). Evaluation of coping styles, metacognition and their relationship with test anxiety. *Journal of Mazandaran University of Medical Sciences (JMUMS)*, *23*, 144–155.
(Guo et al., 2024) Guo, M., Liu, A., Wang, X., & Liu, Y. (2024). The efficacy of a group program integrating compassionate mind training with cognitive behavioral therapy for college students with test anxiety in mainland China. *Mindfulness*, *15*(4), 977–990. https://doi-org.proxy.bsu.edu/10.1007/s12671-024-02342-5.
(Hedlefs-Aguilar et al., 2021) Hedlefs-Aguilar, M. I., Morales-Martínez, G. E., Villarreal-Lozano, R. J., Moreno-Rodríguez, C., & González-Rodríguez, E. A. (2021). Functional measurement applied to engineering students’ test anxiety judgment for online and face-to-face tests. *European Journal of Educational Research*, *10*(3), 1599–1612.
(Hewitt & Stephenson, 2012) Hewitt, E., & Stephenson, J. (2012). Foreign language anxiety and oral exam performance: A replication of Phillips’s “MLJ” study. *Modern Language Journal*, *96*(2), 170–189.
(Higham et al., 2021) Higham, P. A., Zengel, B., Bartlett, L. K., & Hadwin, J. A. (2021). The benefits of successive relearning on multiple learning outcomes. *Journal of Educational Psychology*, *114*(5), 928. https://doi.org/10.1037/edu0000693.
(John Lothes et al., 2021) John Lothes, I. I., Mochrie, K., Wilson, M., & Hakan, R. (2021). The effect of DBT-informed mindfulness skills (what and how skills) and mindfulness-based stress reduction practices on test anxiety in college students: A mixed design study. *Current Psychology: A Journal for Diverse Perspectives on Diverse Psychological Issues*, *40*(6), 2764–2777. https://doi.org/10.1007/s12144-019-00207-y.
(Kausar et al., 2018) Kausar, U., Haider, S. I., Mughal, I. A., & Noor, M. S. A. (2018). Stress levels; stress levels of final year MBBS students and its effect on their academic performance. *Professional Medical Journal*, *25*(6), 932–936. https://doi.org/10.29309/TPMJ/18.4720.
(Thomas & Ozer, 2024) Thomas, C. L., & Ozer, O. (2024). A cross-cultural latent profile analysis of university students’ cognitive test anxiety and related cognitive-motivational factors. *Psychology in the Schools*, *61*(6), 2668–2693. https://doi.org/10.1002/pits.23186.
(Khoshhal et al., 2017) Khoshhal, K. I., Khairy, G. A., Guraya, S. Y., & Guraya, S. S. (2017). Exam anxiety in the undergraduate medical students of Taibah University. *Medical Teacher*, *39*, S22–S26. https://doi.org/10.1080/0142159X.2016.1254749.
(Klug et al., 2021) Klug, K., Tolgou, T., Schilbach, M., & Rohrmann, S. (2021). Intrusions in test anxiety. *Current Psychology: A Journal for Diverse Perspectives on Diverse Psychological Issues*, *40*(5), 2290–2300. https://doi.org/10.1007/s12144-019-0167-x.
(Knoll et al., 2019) Knoll, R. W., Valentiner, D. P., & Holzman, J. B. (2019). Development and initial test of the safety behaviors in test anxiety questionnaire: Superstitious behavior, reassurance seeking, test anxiety, and test performance. *Assessment*, *26*(2), 271–280. https://doi.org/10.1177/1073191116686685.
(Kusturica et al., 2019) Kusturica, J., Hajdarević, A., Nikšić, H., Skopljak, A., Tafi, Z., & Kulo, A. (2019). Neuroenhancing substances use, exam anxiety and academic performance in Bosnian-Herzegovinian first-year university students. *Acta Medica Academica*, *48*(3), 286–293. https://doi.org/10.5644/ama2006-124.269.
(Manchado Porras & Hervías Ortega, 2021) Manchado Porras, M., & Hervías Ortega, F. (2021). Procrastinación, ansiedad ante los exámenes y rendimiento académico en estudiantes universitarios. *Interdisciplinaria*, *38*(2), 242–258.
(Minihan et al., 2021) Minihan, S., Samimi, Z., & Schweizer, S. (2021). The effectiveness of affective compared to neutral working memory training in university students with test anxiety. *Behaviour Research and Therapy*, *147*, 103974.
(Morales-Martínez et al., 2021) Morales-Martínez, G. E., Garcia-Collantes, A., Hedlefs-Aguilar, M. I., Charles-Cavazos, D. J., & Mezquita-Hoyos, Y. N. (2021). Information integration cognitive mechanisms underlying the face-to-face or online statistics test anxiety judgments of engineering students. *European Journal of Educational Research*, *10*(1), 23–37.
(Ne’Eman-Haviv & Bonny-Noach, 2019) Ne’Eman-Haviv, V., & Bonny-Noach, H. (2019). Substances as self-treatment for cognitive test anxiety among undergraduate students. *Journal of Psychoactive Drugs*, *51*(1), 78–84.
(O’Donnell, 2017) O’Donnell, P. S. (2017). Executive functioning profiles and test anxiety in college students. *Journal of Psychoeducational Assessment*, *35*(5), 447–459. https://doi.org/10.1177/0734282916641554.
(Oflaz, 2019) Oflaz, A. (2019). The foreign language anxiety in learning German and the effects of total physical response method on students’ speaking skill. *Journal of Language and Linguistic Studies*, *15*(1), 70–82.
(Palkar et al., 2021) Palkar, D., Panigrahi, S., Shatadal, P., & Mehta, R. (2021). Impact of Jacobson’s progressive muscle relaxation on stress levels of exam-going MBBS students of a medical college in South Gujarat, India. *Journal of Clinical & Diagnostic Research*, *15*(8), 10.
(Patience & Chinyere, 2014) Patience, N. A., & Chinyere, I. N. (2014). Appraisal of the level of test anxiety among tertiary education students: A case of Alvan Ikoku Federal College of Education, Owerri, Imo State, Nigeria. *African Educational Research Journal*, *2*(1), 20–26.
(Pekrun, 2006) Pekrun, R. (2006). The control-value theory of achievement emotions: Assumptions, corollaries, and implications for educational research and practice. *Educational Psychology Review*, *18*, 315–341. https://doi.org/10.1007/s10648-006-9029-9.
(Qin et al., 2021) Qin, Q., Ma, W., & Zhao, L. (2021). Probiotic supplement preparation relieves test anxiety by regulating intestinal microbiota in college students. Disease Markers, 2021, 1–8. https://doi.org/10.1155/2021/5597401.
(Rajiah & Saravanan, 2014) Rajiah, K., & Saravanan, C. (2014). The effectiveness of psychoeducation and systematic desensitization to reduce test anxiety among first-year pharmacy students. *American Journal of Pharmaceutical Education*, *78*(9), 1–7. https://doi.org/10.5688/ajpe789163.
(Ramezani et al., 2016) Ramezani, J., Hossaini, M., & Ghaderi, M. R. (2016). The relationship between test anxiety and academic performance of Nursing and Emergency Medical Technician students. *Education Strategies in Medical Sciences*, *9*(5), 392–399.
(Relojo-Howell & Stoyanova, 2019) Relojo-Howell, D., & Stoyanova, S. (2019). Expressive writing as an anxiety-reduction intervention on test anxiety and the mediating role of first language and self-criticism in a Bulgarian sample. *Journal of Educational Sciences and Psychology*, *9*(1), 121–130.
(Sa’ad Alzboon, 2016) Sa’ad Alzboon, H. (2016). Test anxiety & its relation to perceived academic self-efficacy among Al Hussein Bin Talal University students. *Journal of Education and Practice*, *7*(29), 172–182.
(Savytska, 2019) Savytska, O. (2019). The evaluation of the mental and emotional state of the university students in the situation of exam. *Current Problems of Psychiatry*, *20*(4), 309–317. https://doi.org/10.2478/cpp-2019-0023.
(Schweden et al., 2020) Schweden, T. L. K., Müller, D., Becker, T., Rösch, K., Höfer, S., & Jarczok, M. N. (2020). Evaluation of a brief cognitive behavioral group intervention to reduce depersonalization in students with high levels of trait test anxiety: A randomized controlled trial. *Anxiety*, *Stress & Coping: An International Journal*, *33*(3), 266–280. https://doi.org/10.1080/10615806.2020.1736936.
(Shakil & Saad Ahmed, 2019) Shakil, M., & Saad Ahmed, A. (2019). Motivation systems and test anxiety in undergraduates of COMSATS University Islamabad, Lahore campus. *Pakistan Journal of Clinical Psychology*, *18*(1), 3–16.
(Van Yperen, 2007) Van Yperen, N. W. (2007). Performing well in an evaluative situation: The roles of perceived competence and task-irrelevant interfering thoughts. *Anxiety*, *Stress*, *& Coping*, *20*(4), 409–419. https://doi.org/10.1080/10615800701628876.
(Vaz et al., 2018) Vaz, C. J., Pothiyil, T. D., George, L. S., Alex, S., Pothiyil, D. I., & Kamath, A. (2018). Factors influencing examination anxiety among undergraduate nursing students: An exploratory factor analysis. *Journal of Clinical & Diagnostic Research*, *12*(7), 16–19. https://doi.org/10.7860/JCDR/2018/35878.11817.
(Warnecke & Krohne, 2019) Warnecke, I., & Krohne, H. W. (2019). Design of a guided internet-delivered counseling intervention for test anxiety. *European Journal of Counselling Psychology*, *8*(1), 163–176. https://doi.org/10.5964/ejcop.v8i1.205.
(Woldeab & Brothen, 2019) Woldeab, D., & Brothen, T. (2019). 21st century assessment: Online proctoring, test anxiety, and student performance. *International Journal of E-Learning & Distance Education*, *34*(1). Available online: https://www.ijede.ca/index.php/jde/article/view/1106 (accessed on 1 December 2024).
(Zavery et al., 2021) Zavery, A., Zäch, M., & Bertrams, A. (2021). Test Anxiety in Autistic University Students-Preliminary Results from a German-Speaking Sample. *Brain Sciences*, *11*(3), 390. https://doi.org/10.3390/brainsci11030390.
(Zhang & Qin, 2020) Zhang, X., & Qin, J. (2020). Empirical analysis of the alleviation effect of music on test anxiety of college students. *Revista Argentina de Clínica Psicológica*, *29*(1), 334–339. https://doi.org/10.24205/03276716.2020.45.

## References

[B1-behavsci-15-00331] Abdi H. M., Bageri S., Shoghi S., Goodarzi S., Hosseinzadeh A. (2012). The role of metacognitive and self-efficacy beliefs in students’ test anxiety and academic achievement. Australian Journal of Basic and Applied Sciences.

[B2-behavsci-15-00331] American Psychiatric Association (2022). Diagnostic and statistical manual of mental disorders.

[B3-behavsci-15-00331] Baghei P., Cassady J. C. (2014). Validation of the Persian translation of the Cognitive Test Anxiety Scale. SAGE Open.

[B4-behavsci-15-00331] Campbell D. (2018). Testing faith: An investigation of the relationship between prayer and test anxiety. Social Work & Christianity.

[B5-behavsci-15-00331] Cardozo L. T., Ramos de Azevedo M. A., Morais Carvalho M. S., Costa R., de Lima P. O., Marcondes F. K. (2020). Effect of an active learning methodology combined with formative assessments on performance, test anxiety, and stress of university students. Advances in Physiology Education.

[B6-behavsci-15-00331] Carter R., Williams S., Silverman W. K. (2008). Cognitive and emotional facets of test anxiety in African American school children. Cognition and Emotion.

[B7-behavsci-15-00331] Casari L. M., Anglada J., Daher C. (2014). Estrategias de afrontamiento y ansiedad ante exámenes en estudiantes universitarios [Coping strategies and exam anxiety in college students]. Psicología.

[B8-behavsci-15-00331] Cassady J. C., Viera Gonzaga L. R., Dellazzana-Zanon L. L., Becker da Silva A. M. (2022). Anxiety in the schools: Causes, consequences, and solutions for academic anxieties. Handbook of stress and academic anxiety–Psychological processes and interventions with students and teachers.

[B9-behavsci-15-00331] Cassady J. C., Krägeloh C. U., Medvedev O. N., Alyami M. (2023). The Cognitive Test Anxiety Scale. International handbook of behavioral health assessment.

[B10-behavsci-15-00331] Cassady J. C., Finch W. H. (2020). Revealing nuanced relationships among cognitive test anxiety, motivation, and self-regulation through curvilinear analyses. Frontiers in Education: Educational Psychology (Special Issue on Achievement Emotions in University Students).

[B11-behavsci-15-00331] Cassady J. C., Finch W. H., Heath J. A. (2022). Early assessment of cognitive skills, self-regulated learning skills, and attitudes toward education predict university success at graduation. Journal of Post-Secondary Education.

[B12-behavsci-15-00331] Cassady J. C., Finch W. H., Nicholson O. D., Oedy M. M. (2023). Using self-identified symptoms to reveal profiles and supports: A Mixed-Methods Study. Educational Psychology in Practice: Theory, Research, and Practice in Education.

[B13-behavsci-15-00331] Cassady J. C., Helsper C. A., Quagliano Q. (2024). The collective influence of intolerance of uncertainty, cognitive test anxiety, and academic self-handicapping on learning outcomes: Evidence of a process model. Behavioral Sciences.

[B14-behavsci-15-00331] Cassady J. C., Johnson R. E. (2002). Cognitive test anxiety and academic performance. Contemporary Educational Psychology.

[B15-behavsci-15-00331] Cassady J. C., Pierson E. E., Starling J. M. (2019). Predicting student depression with measures of general and academic anxieties. Frontiers in Education: Educational Psychology.

[B16-behavsci-15-00331] Cizek G. J., Bunch M. (2007). Standard setting: A practitioner’s guide to establishing and evaluating performance standards on tests.

[B17-behavsci-15-00331] Clinton V., Meester S. (2019). A comparison of two in-class anxiety reduction exercises before a final exam. Teaching of Psychology.

[B18-behavsci-15-00331] Dawood E., Al Ghadeer H., Mitsu R., Almutary N., Alenezi B. (2016). Relationship between test anxiety and academic achievement among undergraduate nursing students. Journal of Education and Practice.

[B19-behavsci-15-00331] DiStefano C., Morgan G. (2011). Examining classification criteria: A comparison of three cut score methods. Psychological Assessment.

[B20-behavsci-15-00331] DiStefano C., Motl R. W. (2006). Further investigating method effects associated with negatively worded items on self-report surveys. Structural Equation Modeling.

[B21-behavsci-15-00331] Ergene T. (2003). Effective interventions on test anxiety reduction: A meta-analysis. School Psychology International.

[B22-behavsci-15-00331] Eysenck M. W., Derakshan N., Santos R., Calvo M. G. (2007). Anxiety and cognitive performance: Attentional control theory. European Journal of Personality.

[B23-behavsci-15-00331] Friedman I. A., Bendas-Jacob O. (1997). Measuring perceived test anxiety in adolescents: A self-report scale. Educational and Psychological Measurement.

[B24-behavsci-15-00331] Hembree R. (1988). Correlates, causes, effects, and treatment of test anxiety. Review of Educational Research.

[B25-behavsci-15-00331] Herzer F., Wendt J., Hamm A. O. (2014). Discriminating clinical from nonclinical manifestations of test anxiety: A validation study. Behavior Therapy.

[B26-behavsci-15-00331] Huntley C. D., Young B., Temple J., Longworth M., Smith C. T., Jha V., Fisher P. L. (2019). The efficacy of interventions for test-anxious university students: A meta-analysis of randomized controlled trials. Journal of Anxiety Disorders.

[B27-behavsci-15-00331] Kader A. (2016). Debilitating and facilitating test anxiety and student motivation and achievement in principles of microeconomics. International Review of Economics Education.

[B28-behavsci-15-00331] Kamel O. (2018). The relationship between adaptive/maladaptive cognitive emotion regulation strategies and cognitive test anxiety among university students. International Journal of Psycho-Educational Sciences.

[B29-behavsci-15-00331] Khan Z., Shah F., Iqbal N. (2015). Level of anxiety among two genders appearing for national level test: A comparative study. Journal of Education and Practice.

[B30-behavsci-15-00331] LeBeau R. T., Glenn D., Liao B., Wittchen H.-U., Beesdo-Baum K., Ollendick T., Craske M. G. (2010). Specific phobia: A review of DSM-IV specific phobia and preliminary recommendations for DSM-V. Depression and Anxiety.

[B31-behavsci-15-00331] Liebert R. M., Morris L. W. (1967). Cognitive and emotional components of test anxiety: A distinction and some initial data. Psychological Reports.

[B32-behavsci-15-00331] Lin Y. H. (2021). Clustering and profile analysis on statistics anxiety styles of undergraduate students. International Journal of Intelligent Technologies & Applied Statistics.

[B33-behavsci-15-00331] Lowe P. A., Grumbein M. J., Raad J. M. (2011). Examination of the psychometric properties of the Test Anxiety Scale for Elementary Students (TAS-E) scores. Journal of Psychoeducational Assessment.

[B34-behavsci-15-00331] Lowe P. A., Lee S. W., Witteborg K. M., Prichard K. W., Luhr M. E., Cullinan D., Janik M. (2008). The test anxiety inventory for children and adolescents: Examination of the psychometric properties for a new multidimensional measure of test anxiety for elementary and secondary school students. Journal of Psychoeducational Assessment.

[B35-behavsci-15-00331] McDonald A. S. (2001). The prevalence and effects of test anxiety in school children. Educational Psychology.

[B36-behavsci-15-00331] Morgan A. J., Chittleborough P., Jorm A. F. (2016). Self-help strategies for sub-threshold anxiety: A Delphi consensus study to find messages suitable for population-wide promotion. Journal of Affective Disorders.

[B37-behavsci-15-00331] Munn Z., Peters M. D., Stern C., Tufanaru C., McArthur A., Aromataris E. (2018). Systematic review or scoping review? Guidance for authors when choosing between a systematic or scoping review approach. BMC Medical Research Methodology.

[B38-behavsci-15-00331] Myers S. J., Davis S. D., Chan J. C. (2021). Does expressive writing or an instructional intervention reduce the impacts of test anxiety in a college classroom?. Cognitive Research: Principles and Implications.

[B39-behavsci-15-00331] Nahm F. S. (2022). Receiver operating characteristic curve: Overview and practical use of clinicians. Korean Journal of Anesthesiology.

[B40-behavsci-15-00331] Page M. J., McKenzie J. E., Bossuyt P. M., Boutron I., Hoffmann T. C., Mulrow C. D., Shamseer L., Tetzlaff J. M., Akl E. A., Brennan S. E., Chou R. (2021). The PRISMA 2020 statement: An updated guideline for reporting systematic reviews. BMJ.

[B41-behavsci-15-00331] Pekrun R., Goetz T., Frenzel A. C., Barchfeld P., Perry R. P. (2011). Measuring emotions in students’ learning and performance: The Achievement Emotions Questionnaire (AEQ). Contemporary Educational Psychology.

[B42-behavsci-15-00331] Pintado I. S., Sánchez-Mateos J. D., Escolar-Llamazares M. C. (2016). A stress inoculation program to cope with test anxiety: Differential efficacy as a function of worry or emotionality. Avances en Psicología Latinoamericana.

[B43-behavsci-15-00331] Putwain D. (2025). Understanding and helping to overcome exam anxiety.

[B44-behavsci-15-00331] Putwain D., Chamberlain S., Daly A. L., Sadreddini S. (2014). Reducing test anxiety among school-aged adolescents: A field experiment. Educational Psychology in Practice.

[B45-behavsci-15-00331] Putwain D., Daly A. L. (2014). Test anxiety prevalence and gender differences in a sample of English secondary school students. Educational Studies.

[B46-behavsci-15-00331] Putwain D. W. (2007). Test anxiety in UK schoolchildren: Prevalence and demographic patterns. British Journal of Educational Psychology.

[B47-behavsci-15-00331] Putwain D. W., Daly T., Chamberlain S., Saddredini S. (2016). “Sink or swim”: Buoyancy and coping in the test anxiety and academic performance relationship. Educational Psychology: An International Journal of Experimental Educational Psychology.

[B48-behavsci-15-00331] Putwain D. W., von der Embse N. P., Rainbird E., West G. (2020). The development and validation of a new multidimensional test anxiety scale (MTAS). European Journal of Psychological Assessment.

[B49-behavsci-15-00331] Putwain D. W., Woods K. A., Symes W. (2010). Personal and situational predictors of test anxiety of students in post-compulsory education. British Journal of Educational Psychology.

[B50-behavsci-15-00331] Raffety B. D., Smith R. E., Ptacek J. T. (1997). Facilitating and debilitating trait anxiety, situational anxiety, and coping with an anticipated stressor: A process analysis. Journal of Personality and Social Psychology.

[B51-behavsci-15-00331] Sarason I. G. (1984). Stress, anxiety, and cognitive interference: Reactions to Tests. Journal of Personality and Social Psychology.

[B52-behavsci-15-00331] Sarkin D. B. Ş., Gülleroğlu H. D. (2019). Anxiety in prospective teachers: Determining the cut-off score with different methods in multi-scoring scales. Educational Sciences: Theory & Practice.

[B53-behavsci-15-00331] Segool N., Carlson J., Goforth A., von der Embse N., Barterian J. (2013). Heightened test anxiety among young children: Elementary school students’ anxious responses to high-stakes testing. Psychology in the Schools.

[B54-behavsci-15-00331] Spielberger C. D. (1980). Preliminary professional manual for the test anxiety inventory.

[B55-behavsci-15-00331] Spielberger C. D. (1989). State-Trait Anxiety Inventory: Bibliography.

[B56-behavsci-15-00331] Szafranski D. D., Barrera T. L., Norton P. J. (2012). Test anxiety inventory: 30 years later. Anxiety, Stress, & Coping.

[B57-behavsci-15-00331] Tan S. H., Cassady J. C., Wong J. K. C., Khng K. H., Leong W. S. A systematic review of test anxiety identification and leveling in children and adolescents. Psychology in the Schools.

[B58-behavsci-15-00331] Tan S. H., Pang J. S. (2023). Test anxiety: An integration of the test anxiety and achievement motivation research traditions. Educational Psychology Review.

[B59-behavsci-15-00331] Thomas C. L., Cassady J. C., Finch W. H. (2018). Identifying severity standards on the cognitive test anxiety scale: Cutscore determination using latent class and cluster analysis. Journal of Psychoeducational Assessment.

[B60-behavsci-15-00331] von der Embse N., Hasson R. (2012). Test anxiety and high-stakes test performance between school settings: Implications for educators. Preventing School Failure: Alternative Education for Children and Youth.

[B61-behavsci-15-00331] von der Embse N., Jester D., Roy D., Post J. (2018). Test anxiety effects, predictors, and correlates: A 30-year meta-analytic review. Journal of Affective Disorders.

[B62-behavsci-15-00331] von der Embse N. P., Kilgus S. P., Segool N., Putwain D. (2013). Identification and validation of a brief test anxiety screening tool. International Journal of School and Educational Psychology.

[B63-behavsci-15-00331] von der Embse N. P., Putwain D. W., Francis G. (2021). Interpretation and use of the Multidimensional Test Anxiety Scale (MTAS). School Psychology.

[B64-behavsci-15-00331] von der Embse N. P., Witmer S. E. (2014). High-stakes accountability: Student anxiety and large-scale testing. Journal of Applied School Psychology.

[B65-behavsci-15-00331] Warren M. K., Ollendick T. H., King N. J. (1996). Test anxiety in girls and boys: A clinical-developmental analysis. Behaviour Change.

[B66-behavsci-15-00331] World Health Organization (1992). The ICD-10 classification of mental and behavioural disorders: Clinical descriptions and diagnostic guidelines.

[B67-behavsci-15-00331] Zargarzadeh M., Shirazi M. (2014). The effect of progressive muscle relaxation method on test anxiety in nursing students. Iranian Journal of Nursing and Midwifery Research.

[B68-behavsci-15-00331] Zeidner M. (1998). Test anxiety: The state of the art.

[B69-behavsci-15-00331] Zeidner M., Matthews G., Elliot A. J., Dweck C. S. (2005). Evaluation anxiety. Handbook of competence and motivation.

[B70-behavsci-15-00331] Zohar D. (1998). An additive model of test anxiety: Role of exam-specific expectations. Journal of Educational Psychology.

